# Evaluation of a structured skills training group for adolescents with attention deficit/hyperactivity disorder (ADHD) – study protocol of a randomised controlled trial

**DOI:** 10.1186/s12888-019-2133-4

**Published:** 2019-06-10

**Authors:** Jenny Meyer, Mia Ramklint, Maria Unenge Hallerbäck, Måns Lööf, Johan Isaksson

**Affiliations:** 1 Department of Neuroscience, Child and Adolescent Psychiatry and Psychiatry Unit, Uppsala University, Uppsala, Sweden; 2 Centre for Clinical Research, Child and Adolescent Psychiatry unit, County Council of Värmland, Karlstad, Sweden; 3 Child & Adolescent Psychiatric Clinic, Gävle, Sweden; 4 Department of Women’s and Children’s Health, Karolinska Institute Centre of Neurodevelopmental Disorders (KIND) Centre for Psychiatry Research, Karolinska Institute, Stockholm, Sweden

**Keywords:** ADHD, DBT, Psychoeducation, Group treatment, Therapy, Adolescents, RCT

## Abstract

**Background:**

Attention deficit/hyperactivity disorder (ADHD) has a negative impact on several domains of life. However, there is a shortage of evidence-based non-pharmacological treatments for adolescents with ADHD. A structured skills training group (SSTG) based on dialectical behaviour therapy (DBT) has been used in adult patients with ADHD with some promising results, although the treatment has not yet been adapted or evaluated for adolescents with ADHD. This study protocol describes how this treatment was adapted for an adolescent population and how the efficacy of the SSTG will be evaluated using a randomised controlled trial (RCT) design.

**Methods:**

A sample of 184 adolescents (15–18 years of age) with a diagnosis of ADHD has been recruited from seven child and adolescent psychiatric outpatient units and randomised to either the SSTG or an active control group based on psychoeducation. Measures are conducted weekly during the treatment, as well as 2 weeks before treatment and 2 weeks and 6 months after treatment. The primary outcome measures are ADHD symptoms, functional impairment, quality of life and mindfulness. Secondary outcome measures are symptoms of comorbid psychopathology, perceived stress and sleep problems. This article describes the design, methods and analysis plan for evaluating the efficacy of the SSTG.

**Discussion:**

The study will be the first RCT to examine the acceptability and efficacy of a SSTG based on DBT adapted for adolescents with ADHD. We believe that the study will extend the current knowledge base about psychological treatment for adolescents with ADHD.

**Trial registration:**

ISRCTN registry (ISRCTN17366720). Retrospectively registered May 112,016.

**Electronic supplementary material:**

The online version of this article (10.1186/s12888-019-2133-4) contains supplementary material, which is available to authorized users.

## Background

The presentation of attention deficit/hyperactivity disorder (ADHD) changes across the life span, with younger children displaying more hyperactivity-impulsive behaviours and adolescents and adults exhibiting more symptoms of inattention [1]. During adolescence, emotional lability and substance use become a growing problem, as well as comorbid mood and anxiety disorders [1]. In addition, academic, interpersonal and social problems become more pronounced during this period of life [2–4].

Given the negative impact of ADHD throughout the lifespan, it is imperative to implement treatments that are acceptable and effective for patients of different ages. For children and adolescents, non-pharmacological interventions such as psychoeducation are regarded as the first-line treatment; however, in moderate to severe cases of ADHD a combination of pharmacological and psychosocial treatment is recommended [5, 6]. Most studies on psychosocial treatment for younger age groups with ADHD have included children or parents to children and only a paucity of studies have included adolescents with ADHD [7]. However, because adolescence is a period marked by rapid developmental changes with an increased desire for independence and autonomy [8], it is not evident that treatment methods directed towards children are equally feasible and effective for adolescents. Moreover, compared with children and adults with ADHD, adolescents are more likely to discontinue pharmacological treatment [9] which further underscores the need to develop non-pharmacological treatment options for this group.

Cognitive behavioural therapy (CBT) has proven to be effective in treating children and adolescents with different psychiatric problems, e.g., anxiety and depression [10, 11]. CBT is generally characterised by using concrete goals and individual problem analyses, with focus on how emotions, thoughts and behaviours interrelate within a certain context [12]. A few CBT studies have been conducted in adolescents with ADHD, both delivered individually [13, 14] and in a group setting [15]. Preliminary findings indicate that CBT could be an effective treatment for some adolescents with ADHD, even though more studies are needed to draw any definitive conclusions.

As a further development of CBT, dialectical behaviour therapy (DBT) was originally developed for patients with borderline personality disorder (BPD) and suicidal ideation [16] to specifically target management of interpersonal relations and emotion dysregulation. Within DBT, a strive for balance between change and acceptance within a dialectical framework is emphasised, and techniques such as mindfulness, behavioural analysis, social skills and crises management are continuously practiced in the treatment [17]. There is symptom overlap between ADHD and BPD with difficulties in emotional regulation, impulse control, interpersonal relations, substance abuse and poor self-esteem [18]. Given this overlap in symptoms, a structured skills training group (SSTG) with elements of DBT (as well as CBT) has been developed for adult patients with ADHD [19]. The treatment has thus far yielded mixed results. Using an open study design, the treatment has been associated with a reduction in ADHD symptoms, functional impairment, symptoms of comorbidity and improvement in personal health [19–21]. However, when using a RCT design, a reduction in ADHD symptoms, but not in symptoms of comorbidity, have been reported when compared with a loosely structured discussion group [22]. In the largest RCT performed, only the blinded clinical global impression ratings supported SSTG to be superior to an individual clinical management [23]. These mixed findings highlight the importance of the RCT study design. Current recommendations for first-line treatments for ADHD includes psycho-educative interventions [6]. Therefore, psychoeducation is an appropriate control intervention for future RCT studies.

Although SSTG has not yet been adapted or evaluated regarding its efficacy for adolescents with ADHD, a small pilot study of seven adolescents with ADHD concluded that the treatment was feasible and appreciated by the participants but that the manual needed to be age-adjusted to be more optimal for adolescents [24]. To increase the prerequisite for adolescents to be engaged in and benefit from treatments originally developed for adults it is necessary that the participants understand, perceive and relate to the content. Therefore, adaptations in the language, materials, examples and activities need to be tailored to fit the age group [17, 25]. These adaptations may include the use of visual material [26] to clarify the content, as well as more active and experiential exercises (e.g., role play, discussions and practising skills) to increase engagement [17, 27]. An active approach is likely to be especially important in working with adolescents with ADHD who have difficulties maintaining concentration in teaching situations.

## Aims

The study aims to investigate the efficacy of an age-adapted SSTG based on DBT for adolescents with ADHD in a clinical setting using an RCT design with an active control group based on psychoeducation. The study also aims to investigate whether subgroups (e.g., based on subtype of ADHD and symptoms of comorbidity) respond differently to the treatment, and finally, whether the SSTG is acceptable to adolescents with ADHD. We hypothesise that (i) the SSTG will result in a greater reduction of ADHD symptoms and functional impairment, and an increase in mindfulness and quality of life, than a psychoeducational group treatment, and (ii) the SSTG will lead to a greater reduction of symptoms of comorbidity, perceived stress and sleep problems compared with the psychoeducational group treatment.

## Methods/design

### Study design

The study design is a multicentre RCT with two treatment conditions: 1) A SSTG based on DBT and 2) a psychoeducational group treatment. Participants are randomised to either one of the two manualised interventions, both tailored to be used for adolescents with ADHD and delivered via the local child and adolescent psychiatric outpatient units. The study is planned in accordance with the Standard Protocol Items: Recommendations for Interventional Trials (SPIRIT) 2013 [28], and will be analysed and reported in accordance with the recommendations in consolidated standards of reporting trials (CONSORT) [29]. We strive for transparency and any changes and deviations from the protocol will be reported.

### Participants and procedures

**Fig. 1:**
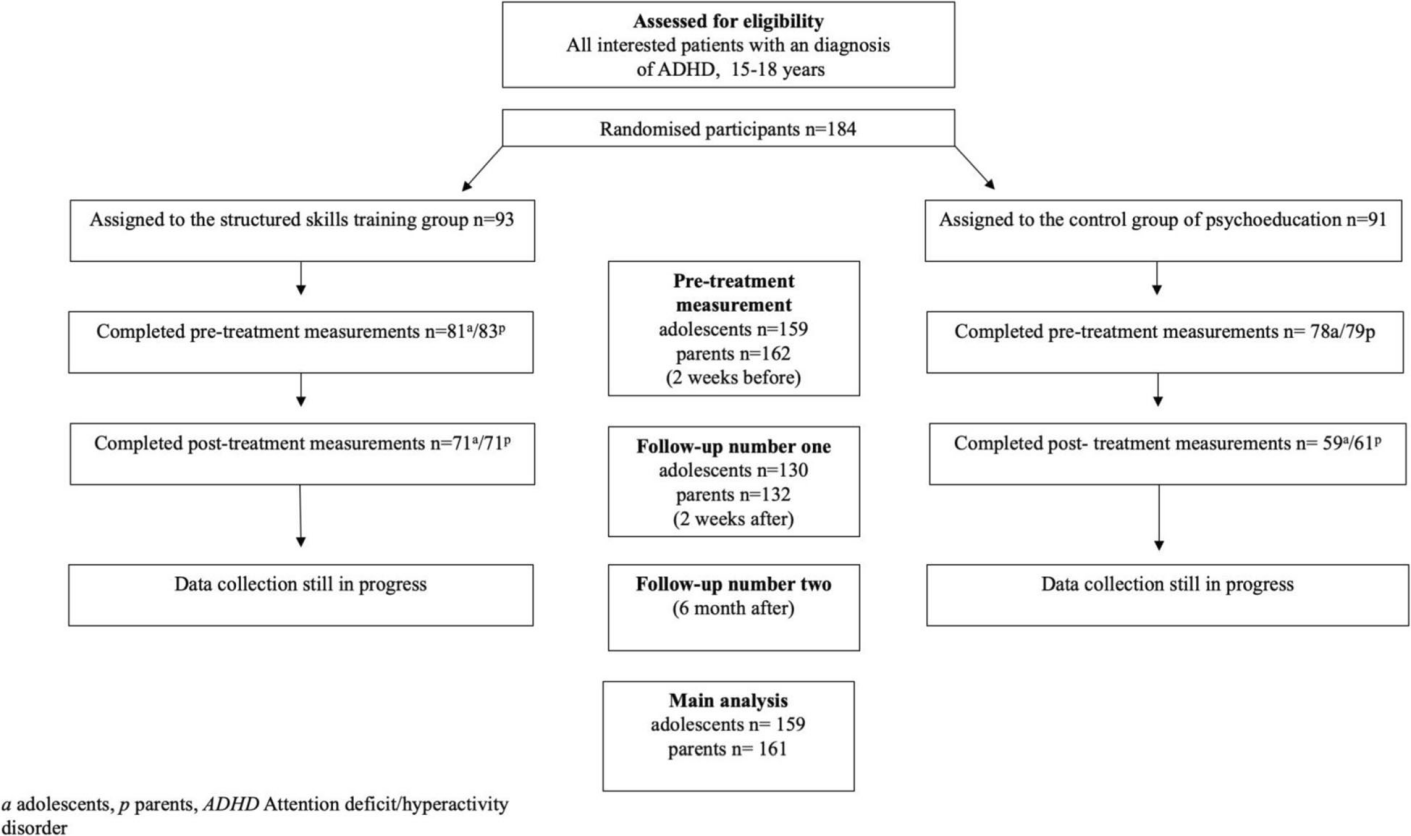
Study flow chart

Recruitment, treatment and data collection are conducted in seven cities in Sweden (Gävle, Falun, Karlstad, Uppsala, Uddevalla, Västerås and Växjö Additional file 1), beginning in 2015, with the last post-treatment measurement expected to be finalised in the spring of 2019. Adolescents (15–18 years of age) from the local child and adolescent psychiatric (CAP) outpatient units with a known diagnosis of ADHD have been informed about the study through flyers in waiting rooms, verbally by the clinical staff, or both. For those who have shown interest, more information has been provided at an extra meeting and the staff have evaluated the patients’ eligibility for the study (see below). Before final inclusion, participants and their parents provided written informed consent. Study participation is voluntary and can be interrupted by the participant at any time. Eligible patients have then been randomised to one of the two treatment conditions. Adolescent self-reports and parental ratings have been collected 2 weeks before the start of the treatment, during the treatment, and are continuously collected 2 weeks and 6 months after conclusion of the treatment. Pre- and post-treatment instruments are administered using an online platform to which only the research group has access and coded data is stored in a secure server. The participating parents are continuously reminded by the research team to complete the questionnaires. When both the adolescent and their parent have completed the last measurement, they will receive two cinema tickets. Initially, the plan was to continue the recruitment until at least 100 patients had completed treatment. In total, 184 patients have been randomised. Of these, 159 adolescents and 162 parents have completed baseline assessments and the last post-treatment evaluations are now continuously being collected (Fig. 1). Those who were randomised but never underwent a baseline assessment were considered external dropouts. Those who completed the baseline assessment, but did not complete the treatment, will be considered internal dropouts.

### Eligibility criteria

To participate in the study the adolescents had to be 15–18 years old, be a patient at one of the designated CAP units and have a diagnosis of ADHD/ADD or ADHD-not otherwise specified according to ICD-10. The diagnosis is validated by using the section for ADHD in the Mini International Neuropsychiatric Interview for Children and Adolescents (MINI-KID) [30]. The assessment was performed by clinical psychologists who were trained in MINI-KID and who also assessed the participants’ eligibility for the inclusion and exclusion criteria. Adolescents were excluded if they showed symptoms of severe depression, suicidality, psychosis or bipolar disorder without a stable medication, a diagnosis of mental retardation, diagnosed organic brain injury, autism spectrum disorder or ongoing substance abuse. Ongoing pharmacological treatment had to be stable and the participants were asked not to change medication during the period of treatment. In addition, the clinical staff were also informed not to change medication or medication dose during the treatment period. Nevertheless, patients will not be excluded if their medication were changed during the treatment period; instead, any changes in medication will be adjusted for in the analyses. The participants were requested not to participate in any other psychological treatment during the treatment period and the parents were requested not to participate in any parent educational programme during this period. The treatment took place at the adolescents’ CAP units and any deterioration of symptoms during the treatment are handled by the units. Any negative reaction could be discussed with the supervisor. Since the treatment is given as part of the regular health care, the patients are covered by the patient injury insurance.

### Randomisation

When eligibility assessment was completed, participants were randomised to one of the two study arms using a computer-generated allocation sequence (https://www.randomizer.org) with separate sequence lists for each treatment centre. The participants were randomly assigned in a 1:1 ratio. Codes were used to ensure information confidentiality and participant anonymity. Moreover, the principal investigator, who had no access to the identity of the participants, performed the treatment allocation based on the codes. Participants were not blinded to the treatment condition.

### Trial arms

#### Structured skills training group (SSTG)

The treatment is an age-adjusted version of a manualised DBT-based group programme originally developed in Germany for adults with ADHD [19, 31] and later translated and evaluated in a Swedish population [22, 32]. The SSTG consists of 14-weekly 2-h sessions. Each session has its own theme (Table 1), including discussions and exercises. The themes are presented to the participants in a workbook and through a PowerPoint presentation. Themes specific for DBT, such as mindfulness, acceptance and behavioural analysis, are continuously practised during the treatment sessions. Each group was led by two therapists, of which at least one was trained in DBT. All of the therapists are educated in the treatment and they discussed the manual in yearly meetings. A clinical psychologist, trained in both CBT and DBT, offered the therapists continuous supervision throughout the study. In addition, the therapists were instructed to video record the group sessions to enable assessment of manual adherence.

**Table 1:** Description of the themes and content of the structured skills training group

Session	Themes
1.	*Introduction:* Introduction and overview of the treatment, psychoeducation about ADHD, strengths, and difficulties with ADHD and introduction of the main themes of treatment. Define goals. Give a home assignment, which is a central part of all sessions.
2.	*Neurobiology and mindfulness I:* Neurobiology of ADHD, emphasising difficulties in executive functions and the possibility to improve functions through practice, e.g. mindfulness training. Introducing mindfulness. Thereafter, mindfulness training is included in all sessions.
3.	*Homework and mindfulness II:* The purpose and importance of homework as a part of the treatment are emphasised. Participants receive tips for how to get the home assignments done.
4.	*Acceptance and mindfulness III*: The balance between acceptance and change is discussed. The concept of “wise mind” and the integration of the emotional mind and the reasonable mind is introduced.
5.	*Chaos and control:* Difficulties in organization and planning are highlighted and strategies with the aim to increase control are introduced.
6	*Emotions:* Education in emotion theory (primary emotions, connection between emotions, thoughts, behaviours, signals and communicatory aspects of emotions, emotions and ADHD). Practice in identifying, observing, and describing emotional signals to regulate and cope with emotions.
7–8.	*Behavioural analysis:* Participants are introduced to behavioural analysis (preceding events/internal and external “triggers”, behavioural responses, and consequences). Strategies to find alternative behaviours are introduced and practiced. Behavioural analysis is applied on own examples and thereafter used throughout treatment.
9.	*Medication, mental illness, and how to increase wellbeing:* Information and discussion about pharmacological treatment for ADHD, documented effects as well as side effects. Symptoms of depression and other emotional problems are discussed, also focusing on treatment, preventive strategies, and emotional regulation.
10.	*Impulsivity, risk behaviour, and addiction:* Symptoms of addiction and other forms of risky behaviours are discussed. Practice in identifying, describing, and regulating impulsive behaviours using mindfulness and behaviour analysis as strategies.
11.	*Stress:* Physiological reactions of stress and the relation between stress and performance are covered. Exercises in identifying and learning about own personal stress are performed. Strategies for stress management are taught.
12–13.	*Self-esteem and relationships:* Differences between self-esteem, self-confidence, and self-respect are clarified, including the impact of ADHD on these areas. Social skills are taught and practiced.
14.	*Retrospect and outlook:* The participants summarize their experience of the group treatment, identify achieved, as well as remaining goals and plan for maintaining progress and how to manage future problems.

A clinical child and adolescent psychologist, trained in both CBT and DBT, adjusted the manual for adolescents based on her clinical experience as well as from the results of the pilot study. The publisher gave us permission to continue to improve the manual for the age group. Further adaptations were discussed and revised in a reference group consisting of five psychologists and one social worker (two were trained DBT therapists and four CBT therapists), all working clinically in child and adolescent psychiatry. The following adaptations were implemented: The language was simplified and examples were adapted to the current age group; some theoretical sections were shortened and clarified; more practical exercises were included; and more examples of tools and skills were presented. Moreover, the number of homework assignments was decreased and no additional literature was recommended. One session was moved to an earlier stage in the programme (the session about emotions was now presented before the session about behavioural analysis) and the time spent on relational skills was increased with additional theory and practice. To make this feasible within the time frame of 14 sessions, the sessions about impulsivity and addiction were merged into one session. Major adaptations were done before the study start and some minor additions were introduced after the first round of treatment to further simplify and clarify the material. After the adaptations were consummated, the study supervisor reviewed the manual to ensure that the original method was preserved. All essential elements were kept.

#### Control treatment

The control treatment (SKILLS) is a manual-based psychoeducational programme designed by JI and ML to be used in group settings. The treatment comprises three 2-h sessions that include information on ADHD symptomatology, strengths and challenges associated with the disorder, information on sleep and food, stress management, problem solving and preparing/structuring daily life routines (Table 2). Moreover, the participants were presented a book about ADHD to read during the treatment programme. DBT-related components (such as mindfulness, behavioural analysis and acceptance) were not included within the control treatment. The groups were led by two therapists, who were: educated in the method, took part in yearly meetings and were offered supervision by ML who is a clinical nurse and a psychotherapist trained in CBT. In addition, the group leaders were instructed to video record the group sessions to enable assessment of manual adherence.

**Table 2:** Description of the themes and content of the psychoeducational treatment (SKILLS)

Session	Themes
1.	*What is ADHD?* Psychoeducation about ADHD including ethology behind ADHD, neurobiology, difficulties and strength with ADHD and examples of famous people with the diagnosis.
2.	*Take charge over your daily life:* Information about how to structure daily life routines and the importance of sleep, food and activity. Introduction to stress management and problem-solving skills.
3.	*Take charge over your ADHD:* A deepening and further work with the problem-solving model, following the structure: Stop and think, get organized, using tools (e.g. mobile apps), using support and coaching.

### Outcome measures

#### Primary outcomes

##### Adult ADHD self-report scale for adolescents (ASRS-A)

ADHD symptoms are assessed using parental and self-ratings on the ASRS-A [33, 34] at pre- (baseline measurement) and post-treatment. Self-ratings were also conducted half-way through the SSTG. The ASRS-A is a modified version of the adult ADHD self-report scale. The instrument has shown promising psychometric properties in clinical populations [34]. The questionnaire contains 18 items corresponding to the diagnostic criteria of ADHD in accordance with the diagnostic and statistical manual of mental disorders, fourth edition (DSM-IV).

##### Impact of ADHD symptoms (IAS)

A six-item questionnaire was constructed for this study to repeatedly assess the impact of ADHD symptoms on the adolescents’ wellbeing. The questionnaire is completed by the adolescents both at pre- and post-treatment but also after each of the sessions. The adolescent responds to the extent their wellbeing has been affected by behaviours and symptoms (e.g., difficulties in regulating emotions and starting/completing assignments on time) related to ADHD during the past week. Each item is answered on a scale from 0 (“Not at all”) to 10 (“Very much”), with higher scores indicating greater impact of the ADHD symptoms on wellbeing. The scale will be validated within the study.

##### Child Sheehan disability scale (CSDS)

Adaptive functioning is measured using the CSDS [35] to assess how much the symptoms interfere with daily functioning (three items for adolescent self-ratings and five for parental ratings). The scale, which has shown good validity in a clinical sample of children and adolescents with psychiatric disorders [35], is a modified version of the Sheehan Disability Scale for adults [36, 37]. The questionnaire is answered before and after treatment by the adolescents and their parents. Self-ratings were also conducted half-way through the SSTG.

##### Global quality of life scale (GQL)

Perceived quality of life is measured with the GQL scale [38], which is a one-item question with a visual analogue scale (VAS). The adolescents answering the question, “How is your life right now?”. The original scale consists of a 10-cm line. However, in the present study measurements were presented in an electronic format in which the 10-cm layout was not possible. Thus, instead we used fixed values ranging from 0 (“The worst imaginable life situation”) to 10 (“The best imaginable life situation”). The GQL is completed by the adolescents before and after treatment as well as after every session. The psychometric properties of the GQL have shown to be acceptable [38].

##### Five facet mindfulness questionnaire (FFMQ)

Level of mindfulness is measured with the FFMQ, which consists of 29 items evaluating aspects of observing, describing, acting with awareness, non-judging of inner experience, and non-reactivity to inner experience [39]. The FFMQ is answered by the adolescents pre- and post-treatment. The construct validity of the scale is supported [39].

#### Secondary outcomes

##### Strength and difficulties questionnaire (SDQ)

Comorbid symptoms are assessed with the SDQ [40], a brief screening questionnaire with 25 items measuring symptoms of emotional and conduct problems, hyperactivity/inattention and peer problems, as well as prosocial behaviour. Moreover, the SDQ consists of an impact supplement targeting level of distress and social impairment. The Swedish version of the SDQ has shown adequate validity and is considered a useful tool for mental health screening in children and adolescents [41]. The SDQ is answered by both adolescents and their parents at pre- and post-treatment.

##### The hospital anxiety and depression (HAD) scale

As the SDQ, the HAD is used to assess comorbid symptoms [42]. The HAD contains 14 items that screen for symptoms of anxiety (seven items) and depression (seven items). This questionnaire is answered by the adolescents at pre- and post-treatment. The scale has been validated and has shown good psychometric properties in a sample of adolescents [43].

##### Pressure activation stress (PAS) scale

Perceived stress is measured with the PAS questionnaire [44]. It consists of 11 items that measure two dimensions of stress: feelings of pressure (e.g., from school demands) and activation (e.g., difficult to relax). The PAS is answered by the adolescents at pre- and post-treatment, as well as half-way through the SSTG. The scale display a promising face validity and good internal consistency [44].

##### Karolinska sleep questionnaire (KSQ)

The KSQ is a self-rating questionnaire measuring sleep problems [45]. In this study we use the seven items corresponding to the subscales “Difficulties falling asleep” and “Difficulties waking up”. The KSQ has shown adequate psychometric properties and is considered a useful tool to assess sleep problems [46].

##### Other measurements

Before and after treatment, parents are asked to report the adolescents’ current medication and dose of medication. During the treatment, the group leaders registered attendance and completed home assignments. During the final session, the adolescents completed an evaluation form specifically constructed for this study to assess the acceptability of the treatment. The evaluation form includes questions on how the participants perceived and responded to the treatment, the content and length of the treatment and if they would recommend the treatment to their peers.

### Statistical methods

#### Statistical analysis plan

The treatments were finalised during 2018 while post-treatment ratings are currently being collected. Analysis of the data will start in the summer of 2019. The statistical analysis will be conducted on all participants who completed the pre- treatment (baseline) questionnaires (*n* = 159 adolescents and *n* = 162 parents). Missing post-baseline data for any of the participants because of the absence of any session or dropout will be handled by multiple imputations using last mean carried forward (LMCF) [47]. A sensitivity analysis will be performed for those who completed the treatments (e.g., attended at least two thirds of the sessions) without using LMCF. Primary and secondary outcome variables will be analysed with repeated measurements in general linear models using the baseline value for respective outcome as a covariate. The primary analysis will be aimed at investigating differences between the two treatment groups in reduction in ADHD symptoms and functional impairment, and increase in mindfulness and quality of life, directly and 6 months post-treatment. Secondary analyses will be aimed at investigating the group treatment effects on reported symptoms of comorbid psychopathology and health-related status. Moreover, the two treatment groups will be compared for treatment acceptability. As separate analyses, we intend to explore any difference in outcome measures based on subgroups of clinical relevance (e.g., subtype of ADHD and symptoms of other psychopathology). We also plan to validate and investigate the psychometric properties of the newly developed questionnaire, IAS, which was constructed to assess the impact of ADHD symptoms on the adolescents’ wellbeing during the treatment intervention. Guidelines for the specific rating scale will be used in the choice of imputation of missing data within the scales. If no guidelines are available, an imputation of the mean of the subscale for that specific participant will be used when less than 20% of the items are missing.

### Sample size

Sample size was calculated using G*Power software version 3.1.9.3 with a power of 0.80 and α = 0.05 (two-tailed). Based on the results from previous studies on CBT/DBT treatment for adults and adolescents with ADHD [15, 21] we expect a reduction on the ASRS-A of about seven points (range is 0–72). A greater reduction of ADHD symptoms in the SSTG of seven points (SD = 12) on the ASRS-A and a greater reduction of four points (SD = 7) on functional impairment as measured by the CSDS would result in an effect size of 0.57 and a sample size of approximately 50 participants per group. Based on pre- and post-treatment measurements using the SDQ from the pilot study, at least 17 adolescents are needed to obtain a reduction of symptoms and 69 adolescents for a reduction of self-rated impairments using the paired sample t-test. Based on these power calculations and what was deemed feasible in the clinical settings, a study population of ≥100 was targeted.

## Discussion

This study will be the first RCT examining the acceptability and efficacy of a SSTG based on DBT for adolescents with ADHD. Recruitment and treatment ended during 2018, the last post-treatment assessments are now being completed. Treatments based on DBT may be of value for adolescents given that the explicit focus on interpersonal relations and emotional dysregulation corresponds to issues of importance during this age [1, 3]. Moreover, the group setting per se may be of importance during this phase of life knowing that adolescence is a time when peers’ behaviour and reasoning have a strong impact on the individual [48]. Thus, the group setting may further reinforce the learning process.

Our study has some limitations. First, the outcome measures are based solely on parental reports and self-reports of the adolescents, which may be subject to reporting bias. Indeed, although rating scales can reliably, validly and efficiently measure DSM-based ADHD symptoms in youths [49], to add a more objective outcome measure, such as assessment by an independent clinician, would have been preferable. Second, a rather large number of the participants (*n* = 32) did not start treatment after being randomised to one of the treatment arms. This pre-treatment drop-out rate may reflect aspects such as not being assigned to the desired treatment or a lack of motivation to participate in the treatment. Given consent to participate may reflect more of an impulsive decision or a willingness to please their parents or the clinician. An attrition analysis will be conducted to further assess potential reasons for attrition and differences between completers and non-completers on the study measures. Third, although we have an active control group, the length of the treatments differs between the two treatment groups, which may have an impact on the outcome measures. More treatment sessions may have a two-fold effect: more attention from the therapist and more time together with the other participants. However, it is not evident that a longer treatment would be perceived as more positive for this group of patients in that participating in a longer treatment is a more time-consuming process. Fourth, some minor adaptations of the treatment manual for the SSTG were conducted after the first round of treatment. However, a comparison between those who received the treatment pre- and post-final adaptations will be calculated for the outcome measures.

The study has some strengths, including being an RCT with an active control setting based on psychoeducation, the recommended first-line treatment. Moreover, data are collected from two information sources, offering the therapists training and supervision and checking adherence to the method. In addition, this study will be carried out at several locations in Sweden, making the sample potentially more representative of the target population. We believe that the study will extend the current knowledge base about psychological treatment for adolescents with ADHD.

## Additional file

Additional file 1: Study Sites. (DOCX 13 kb)

## Abbreviations

ADD: Attention deficit disorder
ADHD: Attention deficit/hyperactivity disorder
ASRS-A: Adult ADHD self-report scale for adolescents
BPD: Borderline personality disorder
CAP: Child and adolescent psychiatric
CBT: Cognitive behavioural therapy
CSDS: Child Sheehan disability scale
DBT: Dialectical behaviour therapy
DSM: Diagnostic and statistical manual of mental disorders
FFMQ: Five facet mindfulness questionnaire
GQL: Global quality of life
HAD: Hospital anxiety and depression scale
IAS: Impact of ADHD symptoms
KSQ: Karolinska sleep questionnaire
LMCF: Last mean carried forward
MINI-KID: Mini International Neuropsychiatric Interview for Children and Adolescents
PAS: Pressure activation stress scale
RCT: Randomised controlled trial
SDQ: Strength and difficulties questionnaire
SSTG: Structured skills training group
