# Supplementary material for: Evaluation of a structured skills training group for adolescents with attention deficit/hyperactivity disorder (ADHD) – study protocol of a randomised controlled trial
Source: BMC Psychiatry. 2019 Jun 10;19:171. doi: 10.1186/s12888-019-2133-4 (PMC6558781; doi:10.1186/s12888-019-2133-4)
Supplement: Supplementary file 1 — Study Sites. (DOCX 13 kb) [file 12888_2019_2133_MOESM1_ESM.docx]

Study cites

- Child and adolescent psychiatric unit, Gävle, Gävleborgs län, Sweden
- Child and adolescent psychiatric unit, Falun, Dalarnas län, Sweden
- Child and adolescent psychiatric unit, Karlstad, Värmlands län, Sweden
- Child and adolescent psychiatric unit, Uppsala län, Sweden
- Child and adolescent psychiatric unit, Uddevalla, Västra Götalands län, Sweden
- Child and adolescent psychiatric unit, Västerås, Västmanlands län, Sweden
- Child and adolescent psychiatric unit, Växjö, Kronobergs län, Sweden
